# Silica Meets Tannic Acid: Designing Green Nanoplatforms for Environment Preservation

**DOI:** 10.3390/molecules27061944

**Published:** 2022-03-17

**Authors:** Fabiana Tescione, Olimpia Tammaro, Aurelio Bifulco, Giovanni Del Monaco, Serena Esposito, Michele Pansini, Brigida Silvestri, Aniello Costantini

**Affiliations:** 1 Institute for Polymers, Composites and Biomaterials of National Research Council (IPCB-CNR), P.le Enrico Fermi 1, 80055 Portici, Italy; fabiana.tescione@ipcb.cnr.it; 2 Department of Applied Science and Technology, Politecnico di Torino, Corso Duca degli Abruzzi 24, 10129 Turin, Italy; serena_esposito@polito.it; 3 Department of Chemical, Materials and Production Engineering (DICMaPI), University of Naples Federico II, P.le Tecchio 80, 80125 Naples, Italy; aurelio.bifulco@unina.it (A.B.); anicosta@unina.it (A.C.); 4 Provincial Department of Caserta, Regional Agency for Environmental Protection of Campania (ARPAC), Via Arena-Centro Direzionale (San Benedetto), 81100 Caserta, Italy; g.delmonaco@arpacampania.it; 5 Civil and Mechanical Engineering and INSTM Unit, University of Cassino and Southern Lazio, Via G. Di Biasio 43, 03043 Cassino, Italy; pansini@unicas.it

**Keywords:** sol–gel synthesis, hybrid silica-based nanoparticles, tannic acid, metal ions adsorption

## Abstract

Hybrid tannic acid-silica-based porous nanoparticles, TA-SiO_2_ NPs, have been synthesized under mild conditions in the presence of green and renewable tannic acid biopolymer, a glycoside polymer of gallic acid present in a large part of plants. Tannic acid (TA) was exploited as both a structuring directing agent and green chelating site for heavy metal ions recovery from aqueous solutions. Particles morphologies and porosity were easily tuned by varying the TA initial amount. The sample produced with the largest TA amount showed a specific surface area an order of magnitude larger than silica nanoparticles. The adsorption performance was investigated by using TA-SiO_2_ NPs as adsorbents for copper (II) ions from an aqueous solution. The effects of the initial Cu^2+^ ions concentration and the pH values on the adsorption capability were also investigated. The resulting TA-SiO_2_ NPs exhibited a different adsorption behaviour towards Cu^2+^, which was demonstrated through different tests. The largest adsorption (i.e., ~50 wt% of the initial Cu^2+^ amount) was obtained with the more porous nanoplatforms bearing a higher final TA content. The TA-nanoplatforms, stable in pH value around neutral conditions, can be easily produced and their use would well comply with a green strategy to reduce wastewater pollution.

## 1. Introduction

Several strategies, such as coagulation, chemical precipitation, chemical reduction–oxidation, ion-exchange, membrane separation and adsorption [1,2,3,4,5,6,7] are commonly used to address the various environmental troubles. Among them, adsorption processes play an outstanding role thanks to their low cost, ease of operation and high effectiveness grade [8,9,10,11,12].

In this view, the manufacture of efficient, valid and cheap adsorbents appears a crucial step in the development of this strategy. The main features of a good adsorbent material are: environmental compatibility, a high number of adsorption sites, good dispersion in water, large surface area, chemical durability, good shape consistency, size controllability and affordability. Furthermore, the design of the adsorbent material must comply with the principles of eco-sustainability [13,14]. In this scenario, plant-derived polyphenols are attracting increasing attention due to their broad chemical versatility, metal chelating and antimicrobial properties [15]. Tannic acid (TA) is a natural glycoside polymer of gallic acid derived from the decomposition of plant biomass. The presence of [16] multiple hydroxyl and phenolic hydroxyl groups (i.e., from catechol and gallic moieties) makes TA suitable for good binding with organic/inorganic/metallic surfaces by covalent and/or non-covalent bond interactions, as well as for the coordination of several metal ions (Cu^2+^, Fe^3+^, Mn^2+^), even in physiological conditions [17,18]. Moreover, TA appears proper for conjugation with an inorganic phase, which is proved to be effective in boosting the intrinsic properties of the organic phase, tuning bio-polymeric supramolecular structures [19,20,21]. The development of TA-based organic–inorganic materials is growing a strong interest as a valid approach to exploit the large reactive potential of these moieties for several technological solutions such as antioxidant products [22,23] and catalytic applications [24,25]. Moreover, Gao et al. synthesized a dopamine functionalized tannic-acid-templated silica nanoparticle for Cu^2+^ removal [26], whereas Javdani et al. [27] investigated the effect of TA as a template of mesoporous silica nanoparticles for the adsorption of iron in acute oral toxicity of ferrous sulphate.

Among inorganic components, due to its intriguing features, silica has been identified as the ideal support for the anchoring of different organic/inorganic materials because of its strong hydrophilicity, tuneable size, shape, porosity, surface chemistry and well-known biocompatibility [28,29,30]. Thus, these characteristic features make silica highly suitable for the development of adsorbent materials. In particular, porous silica particles are largely used in the metal adsorption field and the most exploited strategy involves a post grafting method, which enhances the performances through surface functionalization by amino groups [31,32]. However, this strategy shows several drawbacks, as the amino functionalization leads to high costs related to the precursors and possible high accumulation of NH_2_-groups on the surface, resulting in a sharp drop in the specific surface area [33]. To overcome such limitations, it is necessary to search for low-cost alternatives with high adsorption efficiency. Recently, both the scientific and industrial community are focusing their attention on the design of novel inorganic–organic hybrids offering prominent and promising properties in the areas of adsorbents. It was found that tannic acid can act as a template to tune the adsorption performances of silica by modifying its textural properties. In particular, the ortho-dihydroxyl groups present on tannin compounds are reported to be an efficient complexing agent for their adsorption of heavy metal ions [34,35].

Herein, hybrid TA-silica-based porous nanoparticles were synthesized via a new sol–gel methodology. We also explored the role of tannic acid as both structuring directing agent and green chelating site for heavy metal ions recovery.

The term “sol–gel” has been used since the 19th century to describe all processes involving hydrolysis and condensation reactions, for example, to obtain high-quality SiO_2_ [36,37]. Today, moving beyond this initial connotation, sol–gel processes have become part of the synthesis routine to produce engineering materials [38,39,40,41,42]. In fact, by exploiting features such as low-temperature synthesis and intimate mixing of different species on an atomic scale, the sol–gel technique allows the preparation of porous materials, even in a *one-pot fashion*, reducing reaction steps and with full control of the final product microstructure [36,37,42,43]. In addition, the mild synthesis conditions of wet-chemistry allow preparing sophisticated hybrid organic-inorganic systems, where the synergic characteristics and functionalities of single components extend and improve the properties of the final material [44,45]. The organic component can be coupled to the inorganic one through two main methodologies: by using surface functional groups of preformed inorganic nanoparticles (NPs) or by synthesizing nanoparticles in the presence of the organic phase. Inspired by the last methodology, we designed the sol–gel synthesis of a new hybrid material where the formation of the silica component occurred in the presence of a TA organic phase.

In our procedure, tetrapropyl orthosilicate (TPOS) was chosen as the main source of silica and 3-aminopropyltriethoxysilane (APTS) as the coupling agent. In detail, a very small amount of APTS was first anchored to TA producing a coupled organic-inorganic (TA-APTS) hybrid monomer, actually acting as the driving force in the subsequent generation of silica phase. The resulting hybrid tannic acid-silica (TA-SiO_2_) and bare silica (SiO_2_) nanoparticles, were characterized using Scanning Electron Microscope (SEM), Transmission Electron Microscope (TEM), Fourier Transform Infrared Spectroscopy (FT-IR), Thermogravimetric Analysis (TGA), N_2_ Adsorption/Desorption Analysis at 77 K, Dynamic Light Scattering (DLS) and ζ-Potential. Then, it is well known that industrial, agricultural and domestic activities contribute to the continuous release of heavy metals in water, soil and air, causing a detrimental bioaccumulation with adverse effects on human and animal health [46,47,48]. Damaged or reduced central nervous functions, Alzheimer’s and Parkinson’s disease, metabolic poison and enzyme inhibitors are just a few of the disorders caused by long term exposure to heavy metals [49,50,51,52]. Only a few metals such as copper, chromium, cobalt, magnesium, iron, nickel, manganese, zinc and selenium can have beneficial aspects for plant metabolism in trace amounts, but, above a certain limit (Cu 2.0, Cr 0.1, Fe 0.3, Ni 0.075, Mn 0.4, Zn 5 mg/L, from US EPA/WHO) [53], they can have a negative impact on the quality of soil and its use in food production. Therefore, the removal of heavy metals from water by an appropriate treatment becomes mandatory. Copper ions are the most common pollutants in the wastewater, coming not only from different industrial processes but also from urban stormwater runoff, and their removal from water has been extensively studied by several researchers [54,55,56,57,58]. In Italy, limit values of 0.1 mg/L in surface water and 0.4 mg/L in sewage water are reported in the Legislative Decree 152/2006. In this scenario, preliminary runs of Cu^2+^ removal from water by adsorption on the resulting hybrid nanoparticles materials was performed in order to evaluate their validity in addressing this environmental issue. The adsorption properties of all synthesized NPs have been investigated through Inductively Coupled Plasma Mass Spectrometry (ICP-MS) and UV-vis analysis.

## 2. Experimental Section

### 2.1. Materials

Tetrapropyl orthosilicate (TPOS, 99.999%), 3-aminopropyltriethoxysilane (APTS, 99.999%), copper sulphate pentahydrate, benzotriazole (BTH, Reagent Plus 99%), tannic acid (TA), ammonium hydroxide (28–30% as NH_3_) and 2-propanol (IPA) were purchased from Sigma-Aldrich (Milan, Italy).

### 2.2. TA-SiO_2_ NPs Synthesis

In one tannic acid molecule, five 3,4,5-trihydroxybenzoic acid units act as anchoring sites, since they can be first oxidized to quinone and then coupled with the amino groups of APTS. Different amounts of TA were reacted with the same amount of APTS, to evaluate the effect induced by the different quinone/amino groups ratios on the final NPs architectures. In detail, ammonia (480 μL) and 2-propanol (26 mL) were added to 13 mL of water, followed by the addition of different amounts of tannic acid (30, 60, 120 and 150 mg). After the complete solubilization of TA, APTS (3.70 mM) was added to produce a coupled TA-APTS hybrid monomer, followed by the addition of TPOS (175 mM) dropwise. The obtained systems were kept for 3 h at room temperature, under vigorous stirring, and the obtained TA-functionalized silica nanoparticles (TA-SiO_2_ NPs) were centrifuged and washed three times with distilled water. The four prepared samples were respectively named as 30TA-SiO_2_, 60TA-SiO_2_, 120TA-SiO_2_ and 150TA-SiO_2_ NPs, so as to recall the starting amount of TA used in their manufacture. Table 1 reported the name (Column 1), the quinone/amino groups molar ratio (Column 2) and the nominal amount of TA (Column 3) of all synthesized samples.

The same synthesis route was followed to prepare pristine silica nanoparticles (SiO_2_ NPs) using only TPOS as silica precursor. All prepared samples were stored in aqueous suspension for further use.

### 2.3. Chemical and Physical Characterization of TA-SiO_2_ NPs

The size and the morphology of hybrid nanoparticles were determined with a scanning electron microscopy (SEM) by means of an FEI Quanta 200 FEG (Eindhoven, The Netherlands), equipped with an energy dispersion spectrometer (EDS) (INCAx-act LN2-free detector, Oxford Instruments, Abingdon, UK). Elemental analysis and element mapping for C, O, N and Si atoms were performed on all the samples and performed according to the standard microanalytical procedures. EDS analyses were carried out on the dry samples without any pre-treatment.

The morphological analysis was completed by transmission electron microscopy (TEM) images. Such images were collected using a Tecnai G12 Spirit Twin FEI (Eindhoven, The Netherlands) device with LaB_6_ emission source, equipped with an FEI Eagle 4K CCD camera, under an acceleration voltage of 120-kV. The TEM observations were performed by subjecting the various samples, which were supplied as solid-state powders, to the following procedure. The powders were first dispersed in water and, subsequently, loaded by dip-coating, depositing a drop of dissolved sample, on a grid for TEM type 400 mesh with thin carbon film. After the drying phase, the grid was inserted on a sample holder rod for the TEM characterization.

Dynamic light scattering (DLS) is used to determine nanoparticle size and polydispersity with a Zetasizer Nano-ZS (Malvern Instruments, Worcestershire, UK). Ζ-potential curves of the TA-SiO_2_ NPs were obtained by measuring the electrophoretic mobility as a function of pH by means of electrophoretic light scattering (ELS) on the same instrument. The ζ-potential was measured at r.t. after adjusting the pH gradually by addition of either 0.1 M NaOH or 0.1 M HCl.

Infrared spectra were recorded at room temperature by using an FT-IR spectrometer (Thermo Fisher, Waltham, MA, USA, equipped with a DTGS KBr (deuterated triglycine sulphate with potassium bromide windows), in the region of 4000–400 cm^−^^1^. Each spectrum represents an average of 32 scans using a spectral resolution of 2 cm^−^^1^, corrected for the spectrum of the blank KBr pellet. Samples for FT-IR analysis were prepared by dispersing 1 mg of dried sample in 200 mg of KBr and by pressing the resulting mixture into pellets of 13 mm diameter.

Thermogravimetric analysis of all the synthesized NPs was performed using a Q600SDT apparatus (TA instruments, New Castle, PA, USA), under nitrogen flow, at a rate of 10 °C/min, with temperatures ranging from 40 to 900 °C. This analysis was performed by putting sample masses of approximately 10 mg in a platinum sample pan.

All samples were characterized by N_2_ adsorption at −196 °C on ca. 80 mg sample previously outgassed at 125 °C for 3 h to remove atmospheric contaminants (Quantachrome Autosorb 1, Odelzhausen, Germany). Specific surface area (S_BET_) was calculated according to the Brunauer-Emmett-Teller (BET) method. The Barrett-Joyner-Halenda (BJH) approach was used to calculate the pore size distribution of the sample using the desorption data. X-ray powder diffraction (XRD) patterns were obtained on an X’Pert Phillips diffractometer (Phillips-PANalytical, Almelo, The Netherlands) operating with Cu Kα radiation (1.541874 Å) and equipped with a PIXcel 1D detector (step: 0.026°2θ; time per step: 2 s).

### 2.4. Cu Ions Adsorption Experiments

Cu ions adsorption efficiency was evaluated through both ICP-MS (inductively coupled plasma mass spectrometer, Perkin Elmer model Elan 9000, Shelton, CT, USA) spectrophotometry and UV-vis (Spectrometer Cary 60 UV-vis, Agilent Technologies, Santa Clara, CA, USA).

The adsorption experiments were carried out by using a batch adsorption technique. Firstly, the suspension of a known concentration of TA-silica NPs was sonicated for 10 min at room temperature to remove any aggregates. After that, a suspension amount equivalent to 20 mg of TA-SiO_2_ NPs was added into 50 mL of a Cu^2+^ solution and it was gently stirred at room temperature for 4 h [59,60]. The effect of solution pH on the copper (II) ions adsorption was analysed at different pH (4–10) by adding HCl or NaOH solution.

After the adsorption procedure, the samples were centrifugated (9000 rpm for 30 min) and the supernatant liquid separated from the solid fraction. The amount of Cu^2+^ ions adsorbed by the solid phase (i.e., NPs) was evaluated by ICP-MS, whereas the supernatant phase was analysed by UV-vis technique to achieve a qualitative validation of Cu ions not adsorbed by nanoparticles and, thus, still present in the liquid.

The Cu^2+^ extraction from solid-phase samples was accomplished as follows: the material was sonicated for ~30 min in a 0.08 M HNO_3_ solution, then filtered by using 0.45 μm filters and finally analysed by ICP-MS. Moreover, the copper isotope 63 was employed for all the ICP-MS measurements.

The concentration of the residual Cu^2+^ ions in the supernatants was measured through UV analysis. Optical absorption spectra were recorded using a quartz cell of 1 cm optical path in the 150–800 nm. All spectra were determined at room temperature and corrected for solvent background by calibrating the instrument to the blank solvent. Benzotriazole (BTH) was used to detect copper (II) ions in aqueous solutions through the formation of benzotriazole/copper complex (BTH/Cu^2+^ complex). A certain volume of the supernatant liquid was added into 20 mg/L BTH solution (volume ratio 1:1) and the residual Cu^2+^ ions were evaluated by following the modification of the maximum peak of BTH at 257 nm [60,61,62,63,64].

Finally, the following experimental factors were evaluated: (i) initial Cu ions concentration (from 10 to100 mg/L) and (ii) and the effect of solution pH on the copper (II) ions adsorption. HCl or NaOH solutions were used to modify the pH.

## 3. Result and Discussion

### 3.1. Morphological Characterization

**Figure 1:**
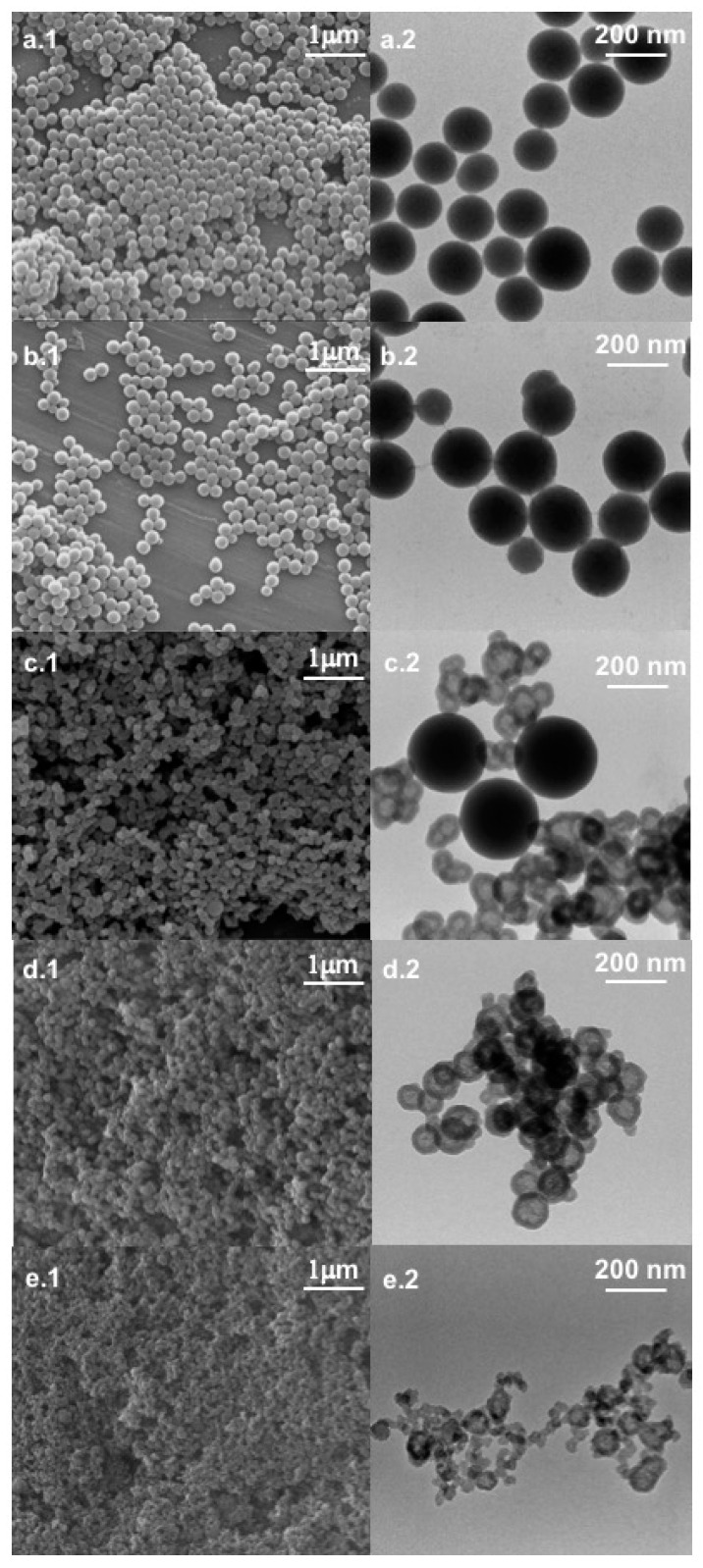
From top to bottom: SEM (left column) and TEM (right column) micrographs of bare silica (**a**), 30TA-SiO_2_ (**b**), 60TA-SiO_2_ (**c**), 120TA-SiO_2_ (**d**) and 150TA-SiO_2_ (**e**) NPs.

The formation of mesostructured nanoparticles as well as their nature and morphology were studied by SEM and TEM analyses. This combination of techniques allows the detection of individual nanoparticles and associated particle-aggregate morphologies. Figure 1 reports electron microscopy images of bare silica nanoparticles (SiO_2_ NPs) and of all hybrid systems synthesized in the presence of different amounts of tannic acid.

**Scheme 1:**
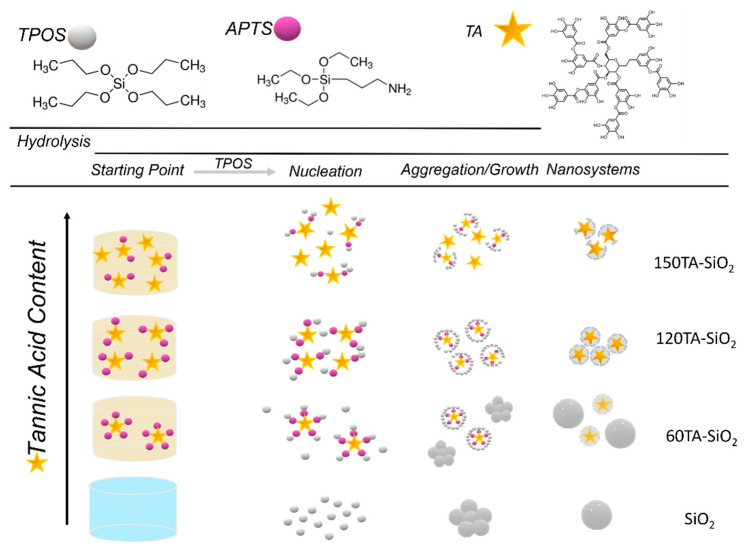
Formation mechanism hypothesized for the proposed synthesis.

Both microscopy analyses revealed a spherical shape for SiO_2_ NPs (Figure 1a), with a mean diameter of about 180 nm. The 30TA-SiO_2_ sample appears quite similar to the bare silica (Figure 1b), but the TEM image points out a fuzzy connection between spherical particles in the typical pearl-necklace structure, which is probably due to the presence of the organic component (Figure 1b). A change in size and morphology can be detected when increasing the TA amount (Figure 1c–e). In particular, 60TA-SiO_2_ shows a double population of particles: more dense spherical NPs (~250 nm in diameter) and aggregates made of small core-shells NPs. We assume that larger spherical nanoparticles are mostly made of silica, while the different densities evidenced by the core-shell structure might be related to the presence of an organic component mainly located in the core, surrounded by a shell characterized by a predominant silica phase. Larger spherical nanoparticles are no longer visible in the micrographs of the 120TA-SiO_2_ sample, mainly composed of clusters of small core-shell NPs. Finally, the 150TA-SiO_2_ sample appears characterized by co-continuous hybrid structures with no well-defined shape (Figure 1e). In view of the above considerations, a possible mechanism related to NPs formation may be proposed (Scheme 1).

Considering the nucleation theory, the first step of NPs generation involves the condensation reaction of hydrated monomers which form primary particles of ~5 nm in diameter. These later aggregate up to the stationary critical size, forming larger nanoparticles [61]. The small number of TA-APTS species used in the synthesis of 30TA-SiO_2_ NPs does not affect the formation mechanism resulting in an NPs population very similar to that of bare silica. In the case of 60TA-SiO_2_, a stoichiometric ratio between amine (-NH_2_) and quinone groups (1:1—mol:mol) is achieved, therefore the amino groups of APTS are all engaged in binding with tannic acid molecules. According to this result, the larger spheres are presumably made of pure silica formed by hydrolysis and condensation reaction of TPOS, whereas it is possible to assume that the concentration of tannic acid is such as to generate coupled TA-APTS hybrid monomers, that act as nucleation sites leading to a second population of core-shell type particles. The hypothesis that there is a limiting concentration of tannic acid to permit the formation of nucleation sites TA-APTS can be supported by the absence of core-shell structures, when a smaller amount of tannic acid was used, keeping constant the other reagent amount. A higher amount of TA (120TA-SiO_2_), resulted in an increasing number of hybrid coupled monomers (see Scheme 1). Therefore, a growing number of homogenous nucleation sites in the starting solution is expected. This behaviour drives towards the formation of solely core-shell particles, formed by the condensation reaction of TPOS, which preferably occurs at coupled TA-APTS species. Finally, at the highest TA content (150TA-SiO_2_), both coupled and free molecules of TA, are expected in the starting solution. Hence, the organic phase can act as a binder and constraining agent in forming organic–inorganic components, limiting NPs growth and producing hybrid clusters with irregular shapes.

**Figure 2:**
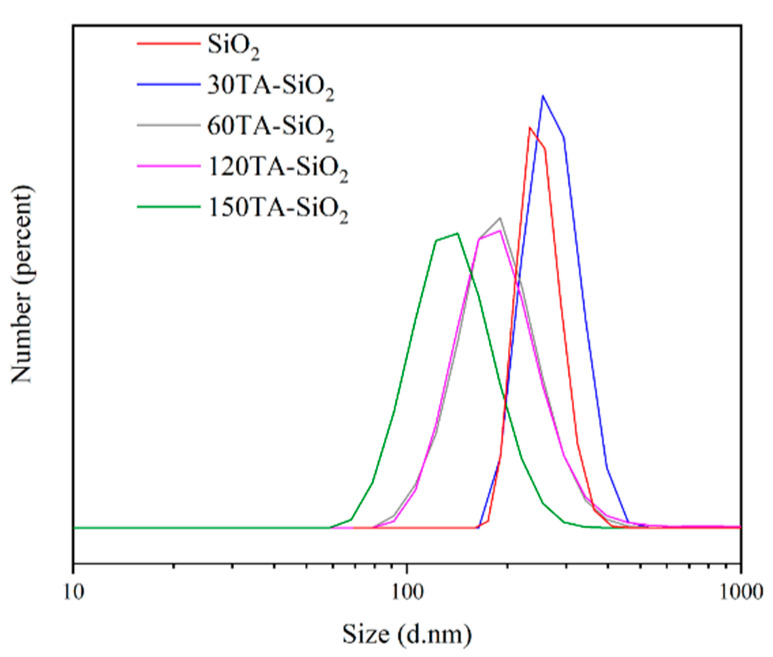
DLS profile of TA-SiO_2_ NPs in deionized water at room temperature.

The DLS distribution presented in Figure 2, points out the significant role of the supposed mechanism in regulating particle size and properties. The curves showed that a decrease in particle size was related to an increase in tannic acid content. For low-content samples (bare SiO_2_ and 30TA-SiO_2_), the curves can be considered almost overlapped with a very low polydispersity value (PDI about 0.02) and higher size value. These data are in good agreement with the hypothesis of spherical monodispersed nanoparticles. In the case of 60TA-SiO_2_ and 120TA-SiO_2_ samples, the curves shifted towards smaller sizes, but the polydispersity index increased (about 0.15). This last observation could indicate the presence of a multicomponent system, which cannot be completely distinguished in its individual parts because of the aggregation effects for smaller particles. This event seems to occur, in particular, for the 60TA-SiO_2_ sample, where electron micrographs show a double population that could be covered by an average aggregation. Finally, for the 150TA-SiO_2_ sample, the average size is even lower, but the presence of a high PDI value could confirm the production of clusters with irregular shapes. The size discrepancy between electron microscopy analysis and DLS measurements is a well-known pitfall, but in our case, the comparison of both results showed the same nanosystems behaviour [62].

### 3.2. Physical–Chemical Characterization

**Figure 3:**
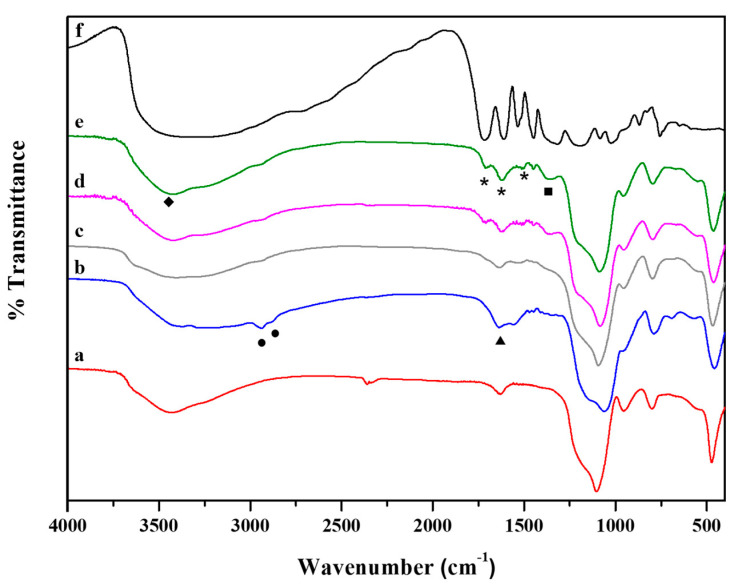
FT-IR spectra of: SiO_2_ (**a**), 30TA-SiO_2_ (**b**), 60TA-SiO_2_ (**c**), 120TA-SiO_2_ (**d**) and 150TA-SiO_2_ NPs (**e**). For the sake of comparison the spectrum of pure TA (**f**) is reported, the highest in the graph.

In Figure 3, FT-IR spectra of hybrid systems are reported together with those of both SiO_2_ and pure TA. The FT-IR spectrum of SiO_2_ NPs (Figure 3a) showed the typical bands of silica gel: (i) 1100 cm^−1^ due to the antisymmetric Si–O–Si stretching vibration mode; (ii) 950 cm^−1^ attributed to Si-O terminal non-bridging vibration; (iii) 800 cm^−1^ and 460 cm^−1^ due to Si–O–Si bond vibration and bending, respectively [63,64]. The NH_2_ bending vibration 1559 cm^−1^ (triangles), emerged in the TA30-SiO_2_ NPs spectrum together with 3190 cm^−1^ adsorptions for NH_2_ groups symmetric stretching [65]. Furthermore, the bands at 2940 cm^−1^ and 2880 cm^−1^ (spectrum b Figure 3, circle) were assigned to the antisymmetric and symmetric stretching of non-hydrolysable alkyl groups of APTS [64]. FT-IR investigation on hybrid samples highlighted that a rising amount of TA (Figure 3c–e) led to the disappearance of primary amino groups signals, coupled with the presence of new bands at 1350, 1505, 1615 and 1707 cm^−1^. The band at 1350 cm^−1^ (squares) was associated with the double bond in the aromatic rings of TA. As reported in the literature, when the hydroxyl groups of the polyphenols were oxidized, the aromatic rings become prone to nucleophile attack by the nitrogen of the primary amino groups [66,67]. This fact resulted in the formation of a secondary amine, as proved by the presence of the new bands at 1505, 1615 and 1707 cm^−1^ (stars), that are mainly visible in the 120TA-SiO_2_ and 150TA-SiO_2_ FT-IR spectra. The absence of the characteristic amide peaks ultimately confirmed that the chemical interaction between TA and the inorganic component occurred through the formation of secondary amines. The presence of TA is detected by the broad band at 3500 cm^−1^ (rhombs) related to the stretching vibration of hydroxyl groups. Commonly, this pronounced band is also present in silica sol–gel derived particles. However, in this case, TA-SiO_2_ samples were synthesized by using two different silica precursors (i.e., APTS and TPOS). This fact results in a lower amount of -OH surface groups because, probably, replaced by non-hydrolysable aminopropyl ones from APTS [45]. Finally, in the hybrid samples spectra, from b to e, the characteristic Si–O–Si stretching vibration in 1000–1300 cm^−1^ region shifted to a higher wavenumber indicating a more crosslinked SiO_2_ network.

**Figure 4:**
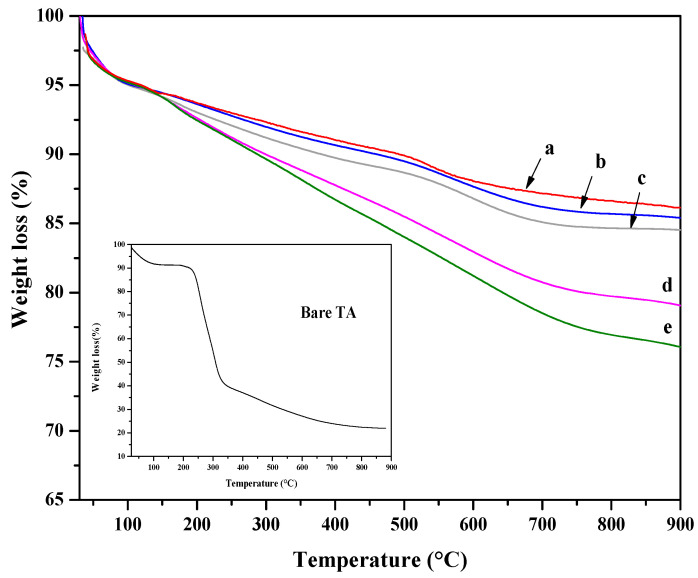
TGA curves of: SiO_2_ (SiO_2_ NPs) (**a**), 30TA-SiO_2_ (**b**), 60TA-SiO_2_ (**c**), 120TA-SiO_2_ (**d**) and 150TA-SI (**e**) NPs. In the inset the TGA of bare TA is reported.

TGA analysis was performed to assess the organic content of the hybrid platforms and thermal stability. In Figure 4, TGA curves of all hybrid nanoparticles are reported together with the ones of SiO_2_ nanoparticles. Moreover, the TGA curve of tannic acid is also shown as an inset in Figure 4. In the TGA curve of pure TA, a first weight loss is observed below 200 °C and attributed to the degradation of tannic acid [68]. The TA decomposition, via decarboxylation, causes a pronounced weight loss in the temperature range of 230–400 °C [69]. TGA curves of SiO_2_ showed a first weight loss of about 5 wt%, at temperatures lower than 150 °C, attributed to the loss of physically adsorbed water. A second weight variation, observed at higher temperatures, was ascribed to the decomposition of residual alkoxide groups and/or dehydroxylation. The TGA curves of 30TA-SiO_2_ and 60TA-SiO_2_ samples resemble that of bare SiO_2_ with a slight increase in weight loss. The trend of the TGA curves is different for 120TA-SiO_2_ and 150TA-SiO_2_ hybrid NPs. It exhibits a continuous weight loss up to 700 °C. This fact seems to suggest higher thermal stability of the organic phase, probably due to the chemical coupling with tIe inorganic component [70,71,72]. The increase in TA in the 120TA-SiO_2_ and 150TA-SiO_2_ samples can account for the greater weight loss.

Elemental analysis was performed on dried nanoparticles to confirm their chemical composition. In Table 2, the elemental weight percent obtained through EDS analysis are reported for all the investigated samples.

It is possible to observe that, the 150TA-SiO_2_ sample showed the largest amount of carbon, due to the highest content of TA in this formulation. This fact is in good agreement with the weight loss data of TG measurements.

**Figure 5:**
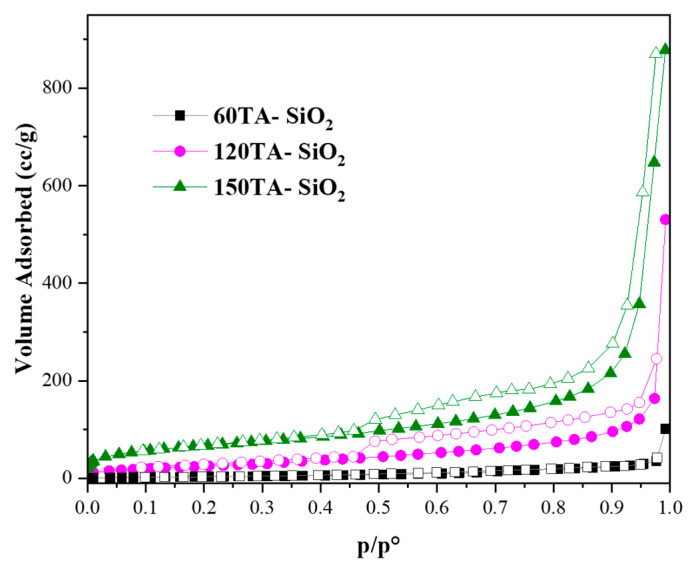
N_2_ adsorption/desorption isotherms at −196 °C of: 60TA-SiO_2_, 120TA-SiO_2_ and 150TA-SiO_2_ NPs. Adsorption and desorption branches were indicated with filled and empty symbols, respectively.

**Figure 6:**
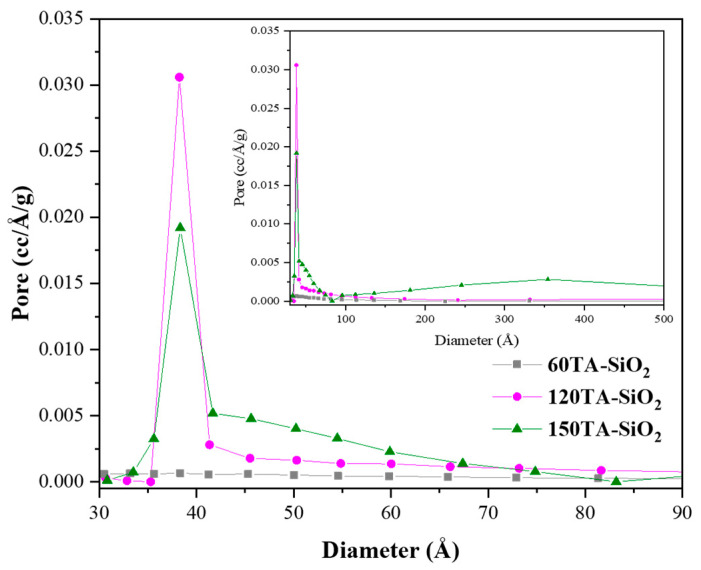
BJH pore size distribution plot derived from the desorption branch of the isotherm for: 60TA-SiO_2_, 120TA-SiO_2_ and 150TA-SiO_2_ NPs. Curves are reported in the small mesoporous range (3–9 nm). In the inset the whole mesopores range (up to 50 nm) is reported.

Hybrid TA-silica samples have been characterized in terms of textural properties. The bare SiO_2_ and 30AT-SiO_2_ nanoparticles are not shown in Figure 5 due to their non-porosity behaviour [73]. In contrast, the reported isotherms, for 60TA-SiO_2_, 120TA-SiO_2_ and 150TA-SiO_2_ NPs systems, show textural properties which appear to be related to the content of tannic acid deployed during the synthesis procedure. The increase in the organic component leads to the formation of porous structures, which exhibit a higher surface area (15 and 237 m^2^∙g^−1^, for 60TA-SiO_2_ and 150TA-SiO_2_ NPs, respectively) and also a rise in the total volume of pores (0.054 and 1.005 cm^3^∙g^−1^, for 60TA-SiO_2_ and 150TA-SiO_2_ NPs, respectively) (Table 3). The isotherm shape of 60AT-SiO2 resembles type III without hysteresis. In this case, the monolayer formation is not identifiable, indicating a non-microporous solid, with a pronounced condensation in inter-particle voids [74]. The shape of the pore size distribution by the BJH method (Figure 6 grey curve) does not show the presence of mesopores, whereas the absence of macropores has been evaluated by the DFT method (data not shown). As far as the 120TA-SiO_2_ and 150TA-SiO_2_ samples are concerned, the fact that the shape of the isotherms seem to be a combination of type III and type II, does not appear to be of simple interpretation. The common type II isotherm, shows increasing adsorption at low relative pressures, indicating the occurrence of micropore filling. As the pressure increases, the monolayer adsorption evolves to multilayer and the adsorbance sharply rises when the *p*/*p*° value is close to 1 (macropore filling). In particular, the adsorption curves rise with a slightly convex shape at values of about *p*/*p*° = 0–0.03, which reveals monolayer physical adsorption and micropore filling. The adsorbance continues to increase slowly until relative pressures *p*/*p*° attain a value close to 1, where a sharp rising occurs, thus indicating the presence of macropores and capillary condensates in the surface of the nanoparticles. The above considerations suggest that the samples 120TA-SiO_2_ and 150TA-SiO_2_ have a complex pore system that includes mesopores and macropores not excluding however micropores. For these samples, moreover, the presence of a type C hysteresis could indicate the presence of open-wedge pores [75]. The pore size distribution, obtained by the BJH method on the desorption branch, is reported in Figure 6. As far as samples 120 and 150 AT-SiO_2_ are concerned, a sharp peak at about 3.8 nm denotes that a large part of their pores are low-range mesoporous. However, the presence of larger pores may be detected, although to a lower extent. Combining these observations with the type of hysteresis found, it is possible to further support the hypothesis of particle formation around a tannic acid core.

### 3.3. Evaluation of Adsorption Capacity

The adsorption capacity was measured by determining the copper amount trapped by nanoparticles using ICP-MS. The results were reported as the amount of adsorbed copper ions per gram of sample (Cu^2+^/TA-SiO_2_ NPs (mg/g)) in Table 4. The data show a comparable content of Cu^2+^ in the case of low TA samples, whereas a remarkable increase in Cu^2+^ is observed for high TA content samples. The general trend suggests a combined effect exerted by both specific surface area and TA component [69,70,71].

**Figure 7:**
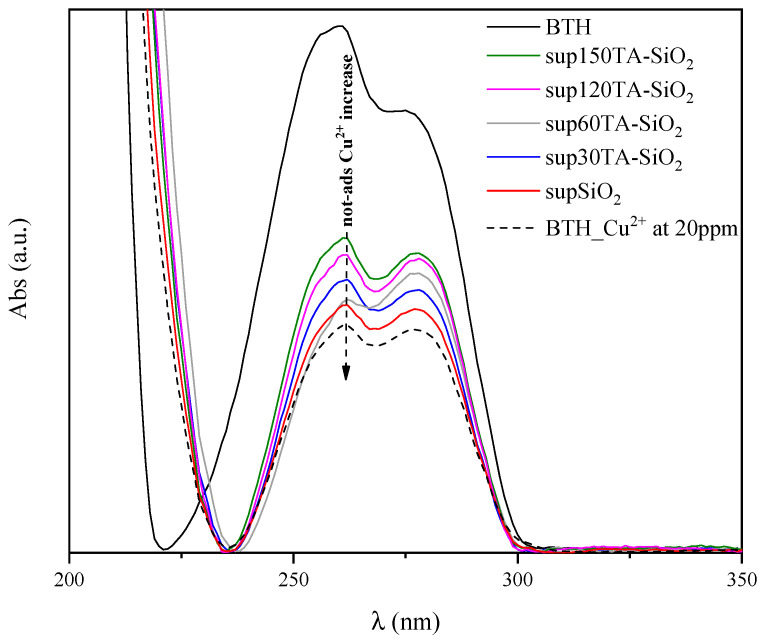
Baseline subtracted UV-vis absorption spectra of BTH; BTH/Cu^2+^ complex and supernatants solutions of SiO_2_, 30TA-SiO_2_, 60TA-SiO_2_, 120TA-SiO_2_ and 150TA-SiO_2_ NPs.

To support ICP-MS data, the adsorption behaviour of TA-SiO_2_ NPs toward Cu^2+^ ions was also qualitatively evaluated by UV-vis spectroscopy measurements. In particular, after the adsorption experiments, all supernatants were recovered to verify the presence of residual copper ions, i.e., the copper not adsorbed by nanoparticles. To this aim, the formation of a benzotriazole–copper complex (BTH/Cu^2+^ complex) was detected by UV-vis analysis and reported in Figure 7 [76,77,78]. In fact, before UV measurement, the samples were diluted two times in a BTH solution (20 mg/L). The curves shown in Figure 7 were baseline corrected.

In the same figure, the spectrum of pure BTH (black curve) was compared with the spectrum of BTH/Cu^2+^ complex obtained with an initial Cu^2+^ ions concentration of 20 mg/L (dotted curve). The formation of the BTH/Cu^2+^ complex resulted in a modification of the pristine BTH curve together with a remarkable reduction in the absorption peak at 257 nm, which is characteristic of the BTH ligand. Moreover, the presence of residual Cu^2+^ ions in the supernatants results in a reduction of this peak. Thus, the larger the reduction, the higher the concentration of residual Cu^2+^ and, hence, the smaller the amount of adsorbed copper onto the nanoparticles [79].

Overall, the trend observed in Figure 7 is in good agreement with ICP-MS results, highlighting that 120TA-SiO_2_ and 150TA-SiO_2_ exhibited the best adsorption performances.

As far as the samples exhibiting the best adsorption ability (120TA-SiO_2_ and 150TA-SiO_2_ NPs) are concerned, the effect of the different Cu^2+^ initial concentrations was investigated and reported in Table 5.

The adsorption capacity significantly increased when the initial copper ions concentration increased up to 20 mg/L. This could be due to the saturation of available active sites on the hybrids nanoparticles above 20 mg/L of copper (II) ions concentration.

In addition, the effect of solution pH on the adsorption of copper (II) ions, for an initial concentration of 20 mg/L, was evaluated and reported in Table 6.

**Figure 8:**
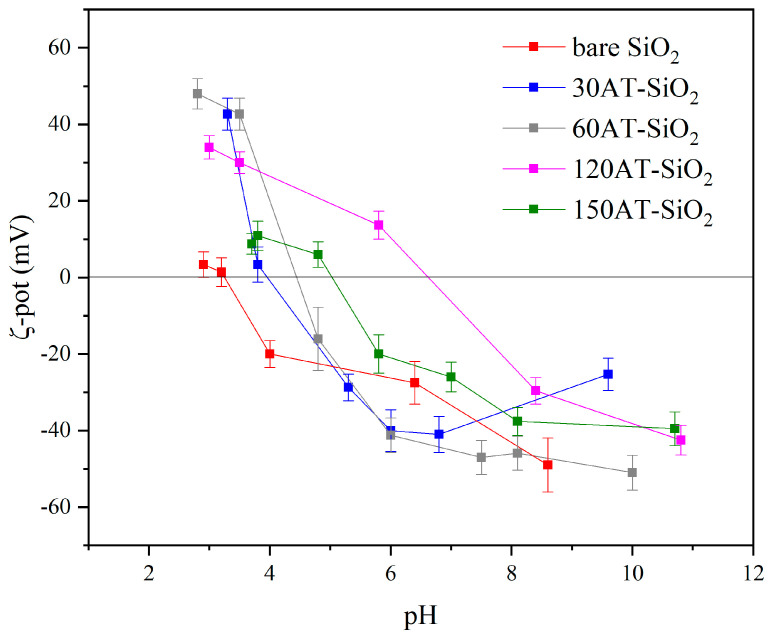
ζ-potential curve of all synthesized samples in deionized water at room temperature.

Both 120TA-SiO_2_ and 150TA-SiO_2_ samples showed that the adsorption capacity is enhanced by the increase in pH. This behaviour could be explained by the different NPs surface charge as a function of pH. It can be seen in Figure 8 that the surface charge for both 120TA-SiO_2_ and 150TA-SiO_2_ NPs was quite different when varying the pH value. The copper ions adsorption is probably favoured by negatively charged surfaces [80]. At low pH, the reduced Cu^2+^ ions adsorption could be attributed to the positively charged surface of TA-SiO_2_ NPs. When the pH value increased from 4 to 7, for both samples, an enhancement in the adsorption capacity was observed, which was larger in 150TA-SiO_2_ NPs, probably due to the higher negative surface charge already observed at pH 7. A further increase in pH value does not influence much the outgrowth in the case of sample 150TA-SiO_2_, while the increase is clear in the case of sample 120TA-SiO_2_, where the charge value moves from about −10mV (at pH = 7) to about −40mV (at pH = 10).

All these results proved that the adsorption of copper ions onto synthesized NPs was highly dependent on the solution pH. Furthermore, the good performances at about pH 7 proved that our systems can be effectively used in the treatment of the real wastewater, which always has pH values close to neutral.

**Figure 9:**
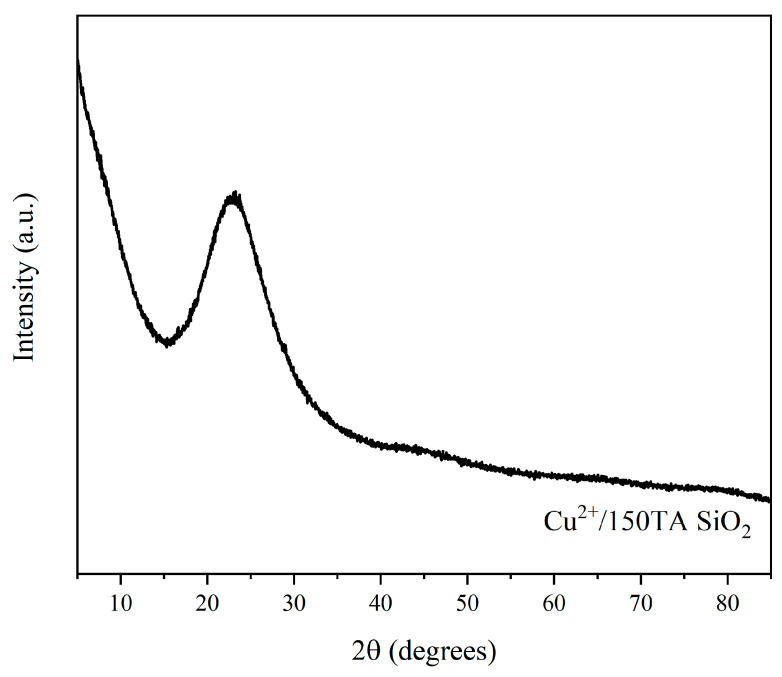
XRD diffractogram of 150TA-SiO_2_ NPs after adsorption experiment with copper ion solution.

Finally, XRD analysis was performed on the 150TA-SiO_2_ NPs after adsorption (Figure 9), to verify the presence of eventual crystalline phases related to the copper component, in view of a secondary application of the hybrid systems. No characteristic crystalline Cu bands were observed, but only a weak signal at 2θ value about 23°, ascribed to amorphous sol–gel-derived silica [81].

## 4. Conclusions

A green and renewable tannic acid biopolymer was successfully employed in the design and synthesis of hybrid TA-silica-based nanoparticles through a one-pot wet-chemistry route. The chemical interaction between the organic and the inorganic components occurred through the formation of secondary amines between aromatic rings of TA and the amino groups of APTS.

TA comes out as a molecule with multifunctional roles:

(I) at a certain concentration it is likely to generate TA-APTS hybrid monomers that act as nucleation sites, thus leading to the formation of core-shell type particles;

(II) it acts as a porogen template, providing a useful tool for varying textural properties;

(III) it acts as a green chelating site for metal ions recovery from aqueous solutions.

In our studies, the high specific surface area, obtained for samples 120TA-SiO_2_ and 150TA-SiO_2_, combined with adequate TA content, resulted in the effective removal of copper ion adsorption.

These findings highlighted the great potential of the proposed synthesis procedure as a valuable strategy to produce sustainable nanoplatforms for metals recovery. Moreover, it was highlighted that the applied sol–gel hybrid methodology may pave the way for the manufacturing of new platforms to be used in the removal of other pollutants, by following an elegant and green approach.

## Figures and Tables

**Table 1:** Quinone/amino groups molar ratio and TA nominal amount used during synthesis.

Sample Label	Quinone/Amino Groups (mol/mol)	TA Nominal Amount (mg)
30TA-SiO_2_	0.6	30
60TA-SiO_2_	1.0	60
120TA-SiO_2_	2.3	120
150TA-SiO_2_	2.7	150

**Table 2:** Elemental weight percent with standard deviation of the samples determined by EDS.

Elemental Weight Percent (wt %)	C	N	O	Si
30TA-SiO_2_	10 ± 2	1.5 ± 1	51 ± 2	38 ± 1
60TA-SiO_2_	12 ± 1	1 ± 0.5	53 ± 0.5	35 ± 1
120TA-SiO_2_	25 ± 2	2 ± 1	47 ± 3	27 ± 2
150TA-SiO_2_	33 ± 3	3 ± 2	44 ± 2	21 ± 2

**Table 3:** Textural properties of 60TA-SiO_2_, 120TA-SiO_2_ and 150TA-SiO_2_ NPs.

Sample Name	Surface Area (m^2^∙g^−1^)	Pore Volume (*p*/*p*^0^ = 0.98)
60TA-SiO_2_	15	0.054
120TA-SiO_2_	97	0.253
150TA-SiO_2_	237	1.005

**Table 4:** Adsorption amount of copper detected by ICP-MS with an initial concentration of Cu^2+^ equal to 20 mg/L (data collected at pH 7).

Sample Name	Cu^2+^/NPs (mg/g)
SiO_2_ NPs	1.90 ± 0.1
30TA-SiO_2_	3.70 ± 0.1
60TA-SiO_2_	2.40 ± 0.1
120TA-SiO_2_	16.0 ± 1
150TA-SiO_2_	26.0 ± 1

**Table 5:** Adsorption amount of copper as Cu^2+^/TA-SiO_2_ (mg/g), detected by ICP-MS, at different Cu initial concentrations.

Initial Concentration of Cu^2+^ (mg/L)	Cu^2+^/120TA-SiO_2_ (mg/g)	Cu^2+^/150TA-SiO_2_ (mg/g)
10	12 ± 1	8.7 ± 0.5
20	16 ± 1	26 ± 1
50	18 ± 1	27 ± 1
100	18 ± 1	26 ± 1

**Table 6:** Adsorption amount of copper as Cu^2+^/TA-SiO_2_ (mg/g), detected by ICP-MS at different pH values.

pH Solution	Cu^2+^/120TA-SiO_2_ (mg/g)	Cu^2+^/150TA-SiO_2_ (mg/g)
4	6.5 ± 0.5	5.6 ± 0.5
7	16 ± 1	26 ± 1
10	27 ± 1	28 ± 1

## Data Availability

Not applicable.
